# Neurologic Alterations Due to Respiratory Virus Infections

**DOI:** 10.3389/fncel.2018.00386

**Published:** 2018-10-26

**Authors:** Karen Bohmwald, Nicolás M. S. Gálvez, Mariana Ríos, Alexis M. Kalergis

**Affiliations:** ^1^Millennium Institute on Immunology and Immunotherapy (MIII), Departamento de Genética Molecular y Microbiología, Facultad de Ciencias Biológicas, Pontificia Universidad Católica de Chile, Santiago, Chile; ^2^Departamento de Endocrinología, Facultad de Medicina, Pontificia Universidad Católica de Chile, Santiago, Chile

**Keywords:** respiratory virus, CNS pathologies, hRSV, Influenza virus, HCoV, hMPV

## Abstract

Central Nervous System (CNS) infections are one of the most critical problems in public health, as frequently patients exhibit neurologic sequelae. Usually, CNS pathologies are caused by known neurotropic viruses such as measles virus (MV), herpes virus and human immunodeficiency virus (HIV), among others. However, nowadays respiratory viruses have placed themselves as relevant agents responsible for CNS pathologies. Among these neuropathological viruses are the human respiratory syncytial virus (hRSV), the influenza virus (IV), the coronavirus (CoV) and the human metapneumovirus (hMPV). These viral agents are leading causes of acute respiratory infections every year affecting mainly children under 5 years old and also the elderly. Up to date, several reports have described the association between respiratory viral infections with neurological symptoms. The most frequent clinical manifestations described in these patients are febrile or afebrile seizures, status epilepticus, encephalopathies and encephalitis. All these viruses have been found in cerebrospinal fluid (CSF), which suggests that all these pathogens, once in the lungs, can spread throughout the body and eventually reach the CNS. The current knowledge about the mechanisms and routes used by these neuro-invasive viruses remains scarce. In this review article, we describe the most recent findings associated to neurologic complications, along with data about the possible invasion routes of these viruses in humans and their various effects on the CNS, as studied in animal models.

## Introduction

### Neurological Manifestations Associated With Respiratory Viruses Infection

Respiratory diseases caused by viral agents are one of the most critical problems in public health, as every year they are responsible for high rates of morbidity and mortality, mainly of young children, the elderly and immunocompromised individuals (Talbot and Falsey, 2010; Tregoning and Schwarze, 2010; Englund et al., 2011). The most common respiratory viruses that affect susceptible population are human orthopneumovirus—previously known as human respiratory syncytial virus (hRSV), influenza virus (IV), coronavirus (CoV) and human metapneumovirus (hMPV; Nichols et al., 2008). The transmission of these viruses is mainly by contact with fomites or suspension droplets (Kutter et al., 2018). All these viruses have in common the ability to produce bronchiolitis and pneumonia, being responsible for large numbers of hospitalizations every winter season (Nichols et al., 2008; Talbot and Falsey, 2010; Tregoning and Schwarze, 2010). Besides respiratory tract infections, these viruses have been associated with neurological clinical manifestations in patients with a severe occurrence of the respiratory disease (Antonucci and Fanos, 2005; Akins et al., 2010; Antonucci et al., 2010; Desforges et al., 2014a; Fok et al., 2015; Algahtani et al., 2016).

Commonly, the invasion of the central nervous system (CNS) and the subsequent pathology have been more studied in infection caused by Japanese encephalitis virus (JEV), Varicella-Zoster virus (VZV), measles virus (MV) and human immunodeficiency virus (HIV), among others (Koyuncu et al., 2013). Nowadays, the interest in increasing the knowledge about the characteristics and mechanisms involved in their neurological manifestations has risen. Neurological abnormalities found in patients with severe respiratory illness have a spread spectrum of clinical signs, being the most reported seizures (Niizuma et al., 2014; Li et al., 2016), status epilepticus (Sweetman et al., 2005; Vehapoglu et al., 2015), encephalopathies (Antonucci and Fanos, 2005; Mizuguchi et al., 2007; Niizuma et al., 2014; Meijer et al., 2016) and encephalitis (Ng et al., 2001; Niizuma et al., 2014; Fok et al., 2015; Table 1). The effects of each respiratory virus mentioned above in the CNS infection will be discussed in detail later.

**Table 1:** Neurological complications and principal laboratory findings associated with respiratory viruses infection.

Respiratory virus	Clinical signs	Laboratory observations	References
Human respiratory syncytial virus (human Orthopneumovirus)	Febrile seizure Convulsion Ataxia Status Epilepticus Meningoencephalitis Cerebellitis Encephalopathy Encephalitis	Viral antibodies in CSF. Viral RNA in CSF (serogroup A and B). Elevated IL-6, IL-8, CCL2 and CCL4 in CSF. Low levels of TNF-α in CSF. Elevated IL-6 and BDNF in CSF correlates with brain damage.	Cappel et al. (1975); Hirayama et al. (1999); Ng et al. (2001); Zlateva and Van Ranst (2004); Otake et al. (2007); Kawashima et al. (2009, 2012) and Morichi et al. (2017)
Influenza	Febrile or afebrile seizures Myelitis Meningitis Encephalitis Guillain-Barre syndrome Acute necrotizing encephalopathy Depression Neuritis Altered state of consciousness Dellirium Abnormal behavior	Pandemic H1N1 isolated from brain post-mortem. Viral material in CSF from patients (H1N1 and H3N2).	Paisley et al. (1978); Salonen et al. (1997); Chiu et al. (2001); Newland et al. (2003); Zlateva and Van Ranst (2004); Sivadon-Tardy et al. (2009); Simon et al. (2013); Xia et al. (2014); Muhammad Ismail et al. (2015); Ruisanchez-Nieva et al. (2017) and Liang et al. (2018)
Coronavirus	Febrile seizures Convulsions Loss of consciousness Encephalomyelitis Encephalitis	Viral detection in brain post-mortem from patients with multiple sclerosis (HCoV-229E, HCoV-OC43). Detection of SARS-CoV and HCoV-OC43 in CSF.	Burks et al. (1980); Arbour et al. (2000); Hung et al. (2003); Lau et al. (2004) and Yeh et al. (2004)
Human metapneumovirus	Febrile seizures Encephalopathy Encephalitis Status epilepticus Altered behavior	Viral RNA in brain post-mortem. Viral RNA in CSF from a patient.	Peiris et al. (2003); Schildgen et al. (2005); Hata et al. (2007) and Sánchez Fernández et al. (2012)

### Routes of Invasion Used by Neurotropic Viruses

For the proper functioning of the CNS, it is essential to maintain homeostasis. Both, the blood-brain and the blood-CSF barriers play an important role in protecting the brain of free passage of unwanted molecules, pathogens and cells (McGavern and Kang, 2011). The blood-brain barrier (BBB) is the first line of defense that prevents the entry of pathogens into the brain, and it is composed by cerebral microvascular endothelium, astrocytes, pericytes and extracellular matrix (McGavern and Kang, 2011; Swanson and McGavern, 2015a). Importantly, the brain microvascular endothelium cells (BMECs) are a cell type found in significant proportions in the BBB; in between these cells are tight junctions (TJ), which controls the barrier permeability (Koyuncu et al., 2013; Miner and Diamond, 2016). Several routes of CNS invasion can be used by viral pathogens, among these are included the hematogenous route—which is the infection of the endothelium or the “Trojan Horse” mechanism—and the peripheral nerves or olfactory sensory neurons (McGavern and Kang, 2011; Swanson and McGavern, 2015b; Dahm et al., 2016).

### The Hematogenous Route for Viral Neuroinvasion

After primary infection, most neurotropic viruses can enter the bloodstream to reach CNS, a process which is called viremia (Gonzalez-Scarano and Tyler, 1987). Once inside the bloodstream, viruses can pass through the BBB by a transendothelial mechanism, which is transcytosis across BMECs and pericytes by endocytic vesicles (Suen et al., 2014). Another transcellular entry method is the infection of endothelial cells, allowing the direct pass across BBB (Koyuncu et al., 2013; Suen et al., 2014). Besides, disruption of BBB permeability by destabilization of TJs allow viral entry into the brain in a paracellular transmigration way (Li et al., 2015; Swanson and McGavern, 2015b). This event is a consequence of a systemic infection that releases inflammatory mediators—such as cytokines and chemokines—besides the matrix metalloproteinase (MMP; Roe et al., 2012). Finally, the “Trojan Horse” mechanism consists in the infection of bloodstream leucocytes—mainly monocytes/macrophages—which can transmigrate via paracellular route, across the permeable BBB into the CNS (Suen et al., 2014).

### CNS Viral Entry Through Peripheral Nerves

The correct working of the organism requires continuous communication between CNS and peripheral tissues. In this process, neurons play an essential role, since these cells innervate the peripheral organs, that can be used by viruses as a gate to enter the CNS (Swanson and McGavern, 2015b). Neurons are polarized; this characteristic allows them to receive, process and transmit signals to other cells (Koyuncu et al., 2013; Swanson and McGavern, 2015a). Some viruses can infect and migrate through the nerve ending which can be sensory or motor (Swanson and McGavern, 2015a). For this purpose, viruses use the motor proteins dynein and kinesins—which are responsible for the retrograde and anterograde neuronal transport (Swanson and McGavern, 2015b).

An alternative route for neuroinvasion is the transport through olfactory neurons (Swanson and McGavern, 2015b). This pathway is an excellent mechanism to access CNS for viruses that enter the body intranasally (Koyuncu et al., 2013). Olfactory nerve has the particularity to be in communication with the nasal epithelium and also with the olfactory bulb, the gateway to the CNS (Koyuncu et al., 2013; Swanson and McGavern, 2015a). This route is commonly used by respiratory viruses that infect the CNS but is not the only one, as it will be discussed later in this review.

## Human Respiratory Syncytial Virus (hRSV)

**Figure 1:**
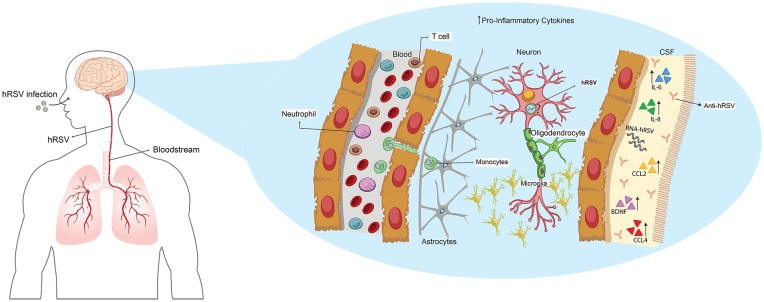
Human respiratory syncytial virus (hRSV) spreads from lungs to the central nervous system (CNS) through hematogenous route altering the local homeostasis. Upon hRSV infection, the virus spreads from the lungs to the brain by hematogenous route. Elevated levels of IL-6, IL8, CCL2, CCL4 and brain-derived neurotrophic factor (BDNF) have been found in cerebrospinal fluid (CSF) from infected patients, along with the detection of antibodies against the virus and viral RNA. It has been suggested that hRSV could infect neurons; however, this was only reported *in vitro* cultures.

The hRSV is an enveloped, negative-sense singled stranded RNA virus, which belongs to the *Mononegavirales* order and has recently been assigned to the *Pneumoviridae* family and the *Orthopneumovirus* genus (Afonso et al., 2016; King et al., 2018). Accordingly, this virus has also been recently renamed human Orthopneumovirus, but for the purpose of these publication we will refer to it as hRSV. The main and most studied pathologies caused by hRSV are bronchiolitis and pneumonia (Antonucci et al., 2010). However, in the past years, extrapulmonary manifestations have been associated with this virus (Eisenhut, 2006). Notably, there is evidence that relates hRSV infection with pathologies such as myocarditis (Esposito et al., 2010), hyponatremia (Hanna et al., 2003), hepatitis (Kirin et al., 2013) and encephalopathy (Ng et al., 2001). In Wallace and Zealley (1970), in a study performed in children with a febrile status, hRSV was detected, and its infection was related to neurological damage. Later, Cappel et al. (1975) detected viral antibodies in cerebrospinal fluids (CSF) of patients that have suffered symptoms of CNS infection such as seizures, convulsions and neck stiffness (Figure 1). One of the most significant findings from this report was that hRSV infection was associated with neurological abnormalities such as encephalitis (Cappel et al., 1975).

A few years later, a case report of three preterm infants which were hospitalized by hRSV-induced bronchiolitis, also presented neurological abnormalities (Morton et al., 1981). Despite these finding, a few years passed until, in Hirayama et al. (1999) reported the case of a 3-year-old child that was hRSV-positive with clinical signs of ataxia. In the CSF, a high number of leucocytes was found; however, they could not detect hRSV by polymerase chain reaction (PCR). The authors concluded that this child manifested a meningoencephalitis with cerebellitis associated with hRSV-infection (Hirayama et al., 1999). In 2001, Ng et al. (2001), performed a retrospective study where clinical data of 487 patients with bronchiolitis by hRSV infection were evaluated. The results of this analysis showed that 1.8% of the children exhibited visible clinical signs of encephalopathy, particularly seizures. Another retrospective investigation that evaluated 226 patients detected that 121 were hRSV-positive and 115 hSV-negative. In the hRSV-positive cohort, about a 6.6% presented seizures; however, this number was similar for the one reported in the hRSV-negative cohort (Kho et al., 2004). In addition to this, it was found that 19.8% of the patients exhibited apnea, but no differences were found when compared with the hRSV-negative cohort (Kho et al., 2004). Importantly, these data support the idea that it is relevant to analyze other symptoms associated with hRSV bronchiolitis carefully.

The first detection of hRSV RNA in CSF was from a 4-month-old boy hospitalized by pneumonia and febrile convulsion (Zlateva and Van Ranst, 2004; Figure 1). In this study, the authors were able to identify that the hRSV strain found belonged to the serogroup B (Zlateva and Van Ranst, 2004). To achieve a better understanding of the effects of hRSV-infection in the CNS, the CSF of an 11-month-old boy that exhibited neurological abnormalities were analyzed, to evaluate the contribution of cytokines in this phenomenon (Otake et al., 2007). The results showed an increase of IL-6 in the CSF but not in serum, which suggests a local effect, implicating that CNS cells—such as astrocytes and microglia—can be the source of these cytokines (Otake et al., 2007; Figure 1). Moreover, an increase of IL-6 was also found in three cases of infants younger than 2-years-old in which it was possible to detect hRSV RNA—serogroup A—in CSF (Kawashima et al., 2009; Figure 1). The authors suggest that their result support the idea of a direct invasion of the CNS by hRSV (Kawashima et al., 2009). Interestingly, the same group found in another cohort of children infected with hRSV the presence of viral RNA, which correlated with low levels of TNF-α in CSF (Kawashima et al., 2012). Also, most of the patients showed an increase in the production of several chemokines such an IL-8, CCL2 and CCL4 which may play an essential role in this disease (Kawashima et al., 2012; Figure 1). The encephalopathies caused by hRSV are classified in four groups: (1) metabolic error type; (2) cytokine storm type; (3) excitotoxicity type; and (4) hypoxic encephalopathies (Morichi et al., 2011). The encephalopathy caused by metabolic error is an abnormality of the brain function that can be reversible and involves an alteration of metabolites. Remarkably, it was found in one of nine patients in this study (Morichi et al., 2011). In the second type of encephalitis, a high increase of several cytokines at systemic levels—which also affect other organs—can be detected. These were reported in only one of the patients (Morichi et al., 2011). In five of the total of patients, excitotoxic encephalopathy was found, which is characterized by febrile convulsion status epilepticus (Mizuguchi et al., 2007; Morichi et al., 2011). Two patients manifested encephalopathies associated with hypoxia, which is a condition that does not include any sign of the others classifications (Morichi et al., 2011). Importantly, hRSV RNA was found in CSF in five of the nine patients analyzed, and the levels of IL-6 were increased only in the patients who exhibited excitotoxic or cytokine storm encephalopathy type (Morichi et al., 2011; Figure 1). Moreover, in all the patients the levels of nitric oxide (NO) were significantly increased independently of the encephalopathy type (Morichi et al., 2011). These results are consistent with the previous report of these authors where they described the finding of hRSV RNA in five of eight patients and also elevated NO levels when compared to influenza-associated encephalopathies (Morichi et al., 2009). Despite the low frequency of neurological complications associated with hRSV-infection, the cases reported exhibit similar profiles, which considers elevated levels of IL-6 in CSF (Miyamoto et al., 2013).

Related to this, Morichi et al. (2017) examined molecular markers in CSF as a prognostic indicator of encephalopathy severity -which includes NO, brain-derived neurotrophic factor (BDNF) and IL-6 (Figure 1). The analysis of these molecular markers was categorized by the encephalopathy type described earlier, in addition to the non-evaluated encephalopathy type (Morichi et al., 2017). Although the authors analyzed a low number of cases, they found that in two patients with cytokine storm encephalopathy, IL-6 and BDNF were significantly elevated, when compared to the control group (Morichi et al., 2017). Moreover, in four patients with non-evaluated encephalopathy, the levels of NO were significantly higher than in the control group (Morichi et al., 2017). The Pediatric Cerebral Performance Category Scale (PCPC) score was used in order to correlate these results to the neurologic prognosis. Only IL-6 and BDNF correlated with PCPC scores, indicating that in more damaged patients, the secretion of these molecules is elevated (Morichi et al., 2017). These tools are nowadays an import advantage in the understanding of the neuropathies associated with hRSV-infection and help to prevent that severe cases lead to death, as was described recently (Xu et al., 2018).

Nowadays, the mechanisms involved in neurological complications due to hRSV-infection remains unknown. Decades ago, researchers adapted the hRSV Long to the brain of newborn mice to study the pathogenesis of this virus in a mice model (Cavallaro and Maassab, 1966; Cavallaro et al., 1967). These authors inoculated the virus intracranially several times and reported that animals exhibited clinical signs of lethargy, ataxia and tremors between the 3rd and 5th-day post inoculation (Cavallaro et al., 1967).

Interestingly, the authors also observed that in a few cases, mice manifested convulsions spontaneously and also died after one or 3 days (Cavallaro et al., 1967). In addition to this, histological analyses showed an association between the extensive necrosis and the liquefaction in the brain with the clinical signs of encephalitis in the mice (Cavallaro et al., 1967). By intracranial inoculation, the authors described that hRSV was not found in others organs and that mice did not exhibit a pulmonary disease (Cavallaro et al., 1967).

During years, there was no report about the relationship between hRSV-infections and CNS pathologies. A few years ago, Li et al. (2006) described, in a study that sought to assess the persistence of infection, the ability of the virus to infect sensory neurons that innervate the lung. These authors hypothesized that hRSV infects not only pulmonary neurons but also that the G-hRSV glycoprotein can interact with the chemokine receptor for CX3CL1 (CX3CR1) expressed in these cells (Li et al., 2006). According to this, they also studied the ability of hRSV to infect primary cortical neuronal cultures and observed, by immunofluorescence, co-localization of N-hRSV protein with neuronal markers. Remarkably, this was not observed when the CX3CR1 was blockade. These results suggest that hRSV can infect neurons *in vitro* at a low percentage (5%) and that it can also infect sensory neurons of the lungs, as reported in culture (Li et al., 2006). This work highlights the fact that hRSV can invade the CNS and infects resident cells which may explain how this virus can cause neurological abnormalities in patients.

To give more insights about this phenomenon, Espinoza et al. (2013) evaluated the neuro-invasive ability of hRSV in a mice model performing an intranasal inoculation that differs in the methodology used in the 60s. Importantly, the authors observed that viral genome and proteins could be detected in the brain of the infected mice, mainly in cortex, hippocampus and ventromedial hypothalamic nucleus (VMH) at 3 days post-infection (Espinoza et al., 2013). Later they evaluated a possible route of entry for hRSV into the brain—the Trojan horse mechanism—by using a blocking antibody for CD49d, which is expressed by leukocytes and is required for transendothelial transmigration of this cells (Espinoza et al., 2013; Figure 1). The results obtained showed a decrease of viral load in the brain of hRSV-infected mice previously treated with anti-CD49d, suggesting that this can be the entry route used by hRSV (Espinoza et al., 2013).

Interestingly, as hRSV proteins were found in the hippocampus—a central zone where the cognitive and behavioral process takes place—alterations in the normal function were evaluated. Both hRSV-infected mice and rats were used for the evaluation of behavior and spatial learning, respectively (Espinoza et al., 2013). Marble burying test was used to evaluate the mechanical digging behavior in rodents, and the data showed that a month after hRSV-infection, these mice exhibited an impairment in this behavior (Espinoza et al., 2013). Moreover, hRSV-infected rats were submitted to the Morris Water Maze test—which evaluates spatial learning—a month after the infection. The data shows that hRSV-infected rats exhibited a delay in their learning capacities, when compared to the control group (Espinoza et al., 2013). Considering these results, it is suggested that hRSV-infection causes behavioral and cognitive sequelae, that have not been described yet in patients.

Recently, an *in vitro* study using neuronal N2a cells as hRSV-infection model—which are a neuroblastoma cell line that can differentiate into cells that possess neuronal characteristic—was performed (Yuan et al., 2018). The data presented by the authors indicated that this virus infects these cells and that viral titers increased up until 96 h post-infection, suggesting that hRSV replicates in this cell line (Yuan et al., 2018; Figure 1). Additionally, they evaluated if toll-like receptor 4 (TLR4) and nucleolin (C23) are able to recognize the F-hRSV protein in N2a cells, as was reported in the literature. Using confocal microscopy, they found that this interaction also occurs in the hRSV-infected cells (Yuan et al., 2018). According to this, they also observed that hRSV-infection increases the protein levels of TLR4 and C23 in N2a cells (Yuan et al., 2018). To evaluate the contribution of infected neurons, in encephalopathies associated with hRSV-infection, the secretion of pro-inflammatory cytokines in the supernatant of N2a-hRSV infected cells was assessed by ELISA. The data obtained showed an increase of IL-6 and TNF-α in N2a hRSV-infected cells when compared to the control cells (Yuan et al., 2018). Despite this new knowledge, there is no *in vivo* evidence that shows whether hRSV infects neurons or other resident cells. More research in this field is required to achieve a better understanding of the mechanism involved in the CNS disease induced by hRSV-infection.

## Influenza Virus

IV is the etiological viral agent most relevant in respiratory tract infections. The Influenza A (IAV), B (IBV), C (ICV) and D (IDV) viruses belong to the *Orthomyxoviridae* family and are the only members of their respective genus, within the Unassigned order (Bouvier and Palese, 2008; Resa-Infante et al., 2010; Su et al., 2017; King et al., 2018). These viruses are enveloped, negative-sense, segmented-stranded RNA and the subtypes of IAV are determined by two structural proteins, hemagglutinin (HA) and neuraminidase (NA; Louten, 2016; Su et al., 2017). There are 18 different HA subtypes (H1-H18) described and at least 11 subtypes of NA (N1-N11; Louten, 2016). Based on this, any combination of HA and NA proteins could be found, being relevant in human diseases the H1, H2 and H3 which are transmitted between individuals (Louten, 2016). In addition to this, when IAV from animals infects naïve human individuals, antigenic shift—a process in which the re-assortment of genes segments from two subtypes of virus—takes place, sometimes leading to IV epidemics, such as the recent H5N1 epidemic (Jang et al., 2009; Louten, 2016; Su et al., 2017). The circulating IAV that are more risk to human health are H1N1, H1N2, H2N2 and H3N2, along with IBV (Louten, 2016; Skowronski et al., 2018).

Associations to respiratory pathologies in IV infections have been described, with neurological complications in both children and adults (Goenka et al., 2014; Popescu et al., 2017; Paksu et al., 2018). Accordingly, clinical signs that have been observed in patients includes encephalitis (Newland et al., 2003), myelitis (Salonen et al., 1997; Zlateva and Van Ranst, 2004; Xia et al., 2014; Ruisanchez-Nieva et al., 2017), meningitis (Liang et al., 2018), seizures (Chiu et al., 2001) and Guillain-Barre syndrome (Sivadon-Tardy et al., 2009). One of the first reports related to the pandemic H1N1 infection in 1918. Therein, the authors describe that the main neurological symptoms were detected in the nerve centers and—time after the infection—manifestations such as depression and neuritis appeared (Turner, 1919).

Considering this background, years later a study of H2N2 (Asian Influenza) showed that neurological complications incidence increased, when compared to H1N1 pandemics and remarkably IAV was isolated post-mortem from the brain of a patient (Kapila et al., 1958). A posterior retrospective study revealed neuromuscular manifestations in 19% of the patients, with a wide range of clinical signs spectrum (Paisley et al., 1978). Moreover, IV was detected in the CSF of one patient from this study (Paisley et al., 1978). Years ago, the detection of IV in CSF from patients with neurological manifestations was rare. Nowadays, there is more and more evidence of the ability of IV to develop neurological damage.

In 2009 a new H1N1 pandemic was the causative agent of high mortality rates and exhibited increased reports of neurological complications. According to this, a retrospective study of the clinical files of 55 patients infected with H1N1 detected a 50% of visible neurological symptoms (Asadi-Pooya et al., 2011). In this cohort, the most frequent neurological sign reported was headache −35% of the patients—and a few were diagnosed with severe neurological complication −9% of the patients (Asadi-Pooya et al., 2011). Another study performed in Malaysia—that collected clinical data from pediatric hospitals during the 2009 pandemic—reported that 8.3% of children under 5 years old presented neurological manifestation, among which the 66.9% of them manifested febrile seizures (Muhammad Ismail et al., 2015). Importantly, 13.6% and 3.9% of children exhibited influenza-associated encephalitis and acute necrotizing encephalopathy (ANE), respectively (Muhammad Ismail et al., 2015). However, there was no detection of IAV genetic material in the four CSF samples available, whereas brain-imaging showed that a few patients exhibited alterations such as cerebral edema and ANE, besides the three cases where the neurological sequelae were permanent (Muhammad Ismail et al., 2015).

**Figure 2:**
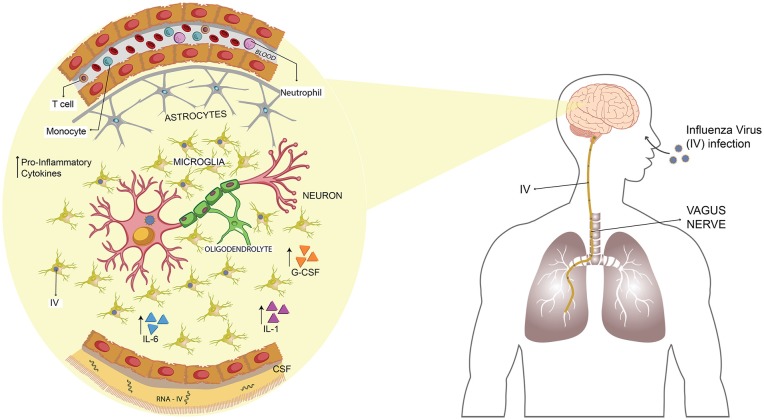
Influenza virus (IV) spreads from the lungs to the CNS through the vagus nerve promoting an inflammatory state. Upon infection of IV, the virus reaches the lungs and, from there, it can spread into the CNS by transneural route, through the vagus nerve. Once set on the brain, it induces the secretion of several pro-inflammatory cytokines such as IL-1, IL-6, IL-8 and G-CSF. Viral RNA has been detected in CSF of infected patients, and also microglial apoptosis has been described.

According to this information, Landau et al. (2011) described that in a cohort of 74 hospitalized children, 19% of them presented neurologic complications mainly seizures. Only one patient from this study was diagnosed with transverse myelitis and presented permanent sequelae (Landau et al., 2011). In addition to this, a fatal case attributed to the H1N1 pandemic infection was reported, and the clinical finding showed that the cause of death was an intracerebral thrombosis and hemorrhage with presence of the virus in the brain, but not in lungs or CSF (Simon et al., 2013; Figure 2).

The knowledge not only came from epidemic IAV, as these neurological signs also have been described for seasonal IAV. The H3N2 and H1N1 seasonal IAV have also been associated with neurological manifestations. According to this, a study described in 21 patients of a wide range of age with neurological alterations showed that the primary clinical sign was encephalitis and about 50% of the patients have sequelae (Steininger et al., 2003). Moreover, although in this study the detection methodology for IV genetic material detection in CSF was improved, only one sample was positive (Steininger et al., 2003; Figure 2). In another approximation to understand the etiologic agent causing myelopathy post-influenza-like syndrome, CSF obtained from a patient with this disease was inoculated in several cell lines, previously reported to be permissive for the growth of this virus (Paiva et al., 2013). Importantly, seasonal IAV H3N2 was detected in MDCK cells, identified as the cause of the neurological symptoms of the patient (Paiva et al., 2013). Besides the main symptoms reported for seasonal H1N1, H3N2 and IBV, altered state of consciousness are consistently detected with seizures, in patients infected with pandemics IAV subtypes (Newland et al., 2003; Popescu et al., 2017; Paksu et al., 2018). Both in this and in others studies, it has been difficult to correlate clinical signs such as CSF pleocytosis with neurological manifestation; mainly due to the low number of patients that exhibit pleocytosis (Paksu et al., 2018). However, the presence of neuroradiological diagnosis suggests that the phenotype observed in patients may be a cause of direct viral invasion into the CNS (Paiva et al., 2013; Paksu et al., 2018). Besides the neurological signs described above, a study in Japan reveals that patients with neurological manifestations also had mild impairment of consciousness, typically delirium or hallucinations and abnormal behavior among others, which belongs to the neuropsychiatric disorders (Manjunatha et al., 2011; Mizuguchi, 2013). Accordingly, there is still necessary to perform more studies about the incidence of these neuropsychiatric manifestations, besides the direct evidence of this association with IV infection.

Highly pathogenic strains of IAV have been used as a model to test the mechanisms involved in the CNS abnormalities caused by IV-infection in humans. Using an H5N3 IAV that initially originated from a water bird and was adapted into chickens to increase its virulence, Shinya et al. (1998) inoculated mice intranasally and evaluate its neurovirulence. The histological data collected exhibited that IAV caused non-suppurative encephalitis. Remarkably, they could also recover IAV from the brain until day 7 post-infection (Shinya et al., 1998). Years later, this group performed experiments using the same virus to elucidate the route used for entry into the CNS. Mice were infected intranasally or intravenously, and only the first group exhibited bronchitis and viral detection in the mucosal epithelium of the trachea to the bronchiole (Shinya et al., 2000). Later, when they evaluated the brain histology, they observed that in addition to the non-suppurative encephalitis, there was an infiltration of macrophages and lymphocytes (Shinya et al., 2000). Finally, the most critical finding was that viral antigens were detected in the vagal and trigeminal ganglia at day 3 post-infection (Figure 2). This event was preceded by the early infection of the nasal cavity, trachea and lungs (Shinya et al., 2000).

Additionally, the hypothesis of the transneural invasion was corroborated in a study performed by Matsuda et al. (2004), which showed that IAV reaches CNS mainly via the vagus nerve (Figure 2). Moreover, through an *in vitro* assay—utilizing neuron cultures in a compartmentalized system—authors suggested that the mechanism of the neurotropic H5N3 to reach CNS is a retrograde axonal transport (Matsuda et al., 2005). Another research using the H5N1 IAV (Hong Kong/483/97) also found that both RNA and viral antigens were detected, first in the vagal and trigeminal ganglia, then later in the brainstem (Park et al., 2002).

The next step in the research was to evaluate in more detail the effects of IV in the brain of challenged mice. In this context, Jang et al. (2009, 2012) observed that mice challenged intranasally with H5N1(Vietnam/1203/04), viral detection in CNS was positive at 3 days post-infection and that the virus can infect neurons and microglia but not astrocytes (Figure 2). In addition to this, they report that H5N1 infection promotes microglial apoptosis, inducing an inflammatory state, which lasts up to 90 days post-infection, similarly to the idiopathic Parkinson’s disease in human (Jang et al., 2009). Moreover, the authors show a loss in dopaminergic neurons in about a 17%, that began as a local immune response that could contribute to CNS disease as is described in humans (Jang et al., 2009). Later research demonstrated that the recovery of the neuronal lasted until 90 days post-infection and that, mainly in the substantia nigra pars compacta (SNpc), the profile of cytokines was altered due to the H5N1 infection (Jang et al., 2012).

Interestingly, IL-13 showed an early induction in the acute phase of the viral infection and then decreased rapidly, to eventually increase after 60 days post-infection (Jang et al., 2012). Moreover, GM-CSF, another cytokine, was not detected in the acute infection, but increased at the same time as IL-13 increased, whereas cytokines such an IL-1, IL-6 and G-CSF, among others, were only detected until 21 days post-infection (Jang et al., 2012; Figure 2). All the data suggest that the local immune, response mediated mainly by microglia, promotes neurons death and protein aggregation, inducing the development of neurodegenerative diseases (Jang et al., 2012). This phenomenon was also observed for H1N1 (CA/09), which promotes the microglial activation mainly in the SNpc and the hippocampal dentate gyrus, however, this virus is not neurotropic (Sadasivan et al., 2015). Additionally, this virus is not able to induce the disruption of BBB which is consistent with the absence of immune cells infiltration into the CNS (Sadasivan et al., 2015).

The IV infection has also been related to neuropsychiatric disorders. Yu et al. (2014) used the neonatal model to evaluate whether IV infection might cause alterations in normal brain functions. Unlike other studies described above, in this one, IV was administered intraperitoneal, as the primary focus of the research was to evaluate the systemic spread of the mouse-adapted H1N1 (NWS/33; Yu et al., 2014). The results showed viral detection in the hippocampus, cerebellum and cerebral cortex among other zones. In addition to this, in infected brain zones, neurons and astrocytes underwent apoptosis, which is consistent with neuroinflammation accompanied by gliosis (Yu et al., 2014).

Moreover, viral RNA was detected in CSF from adult mice, but this does not discard the possibility that in neonates, this also occurs and that IAV furthermore invades the CNS by crossing the blood-CSF barrier (Yu et al., 2014). According to these findings, specifically the detection of the viral RNA in the hippocampus, Hosseini et al. (2018) recently evaluated three different mouse-adapted IAVs: two non-neurotropic virus H1N1 (PR8) and H3N2 (maHK68); one neurotropic virus H7N7 (rSC35M). As they expected, no viral particles were found in the brain of H1N1-infected mice, although a few amounts of viral particles were found in H3N2-infected mice and viral detection was evident in several brain zones in H7N7-infected mice (Hosseini et al., 2018). Therefore, no signs of pathological changes were detected in the brain of H1N1- and H3N2-infected mice, but in the H7N7-infected mice, there was a moderate immune cell infiltration and zones with gliosis (Hosseini et al., 2018).

Interestingly, when they evaluated the effects of IAV infections in the behavior, no virus has affected the mice anxiety or locomotor activity whereas at 30 and 120 days post-infection, however, H3N2 and H7N7-infected mice showed an impairment of spatial learning and memory (Hosseini et al., 2018). This work proves that, unlike what is reported by Jurgens et al. (2012)—which perform cognitive test and neuron morphology experiments during the acute phase of the infection—the H1N1 infection does not lead to long-term impairment in spatial memory nor affects the neuron morphology (Hosseini et al., 2018). The H3N2 subtype is not able to replicate in the CNS; however, it is capable of increasing the levels of TNF-α in the hippocampus and also increase the number of microglia (Hosseini et al., 2018; Figure 2). On the other hand, H7N7 not only increases the level of IFN-γ and TNF-α in the CNS but also alters the long-term potentiation (LTP) and disrupts the permeability of the BBB, promoting a stronger inflammatory immune response than H3N2 (Hosseini et al., 2018). Based on all these data, the most important conclusion is that it is necessary to know the immune response promoted by IVs, as it has been proven that long-term alteration can be caused without CNS viral replication.

Importantly, all the knowledge that we have today about IV infection allow us to be more prepared to diagnose and treat more efficiently patients with this infection.

## Coronavirus

CoV is a group of viruses that belong to the *Coronaviridae* family and the *Nidovirales* order. Accordingly, there are four genera of CoV within the *Coronavirinae* subfamily: *AlphaCoV* (ACoV), *BetaCoV* (BCoV), *DeltaCoV* (DCoV) and *GammaCoV* (GCoV; King et al., 2012, 2018). Their name proceeds from their characteristic crown-shape and is responsible for a wide range of respiratory and enteric diseases in several hosts, such as rodents, cats, pigs and humans (Desforges et al., 2014b). There are several Human CoV (HCoV) described as pathogenic in humans, among which are included HCoV-OC43, HCoV-229E, Middle East respiratory syndrome CoV (MERS-CoV) and severe acute respiratory syndrome CoV (SARS-CoV), all of them with their respective different genotypes (Gaunt et al., 2010; Cabeça et al., 2013; Matoba et al., 2015). Remarkably, neurotropic and neuro-invasive capabilities have been described in several of their hosts, including humans among them, leading to symptoms such as multiple sclerosis (MS) and encephalomyelitis (Lau et al., 2004; Yeh et al., 2004; Zlateva and Van Ranst, 2004; Talbot and Falsey, 2010). However, the capacity of CoV to infect CNS in humans is not well characterized, with their detection in these samples performed mainly by detection of viral RNA, exhibiting persistent infection (Arbour et al., 2000; Desforges et al., 2014a).

CoVs are enveloped viruses with a positive non-segmented single-stranded RNA genome of about 30 Kb of length, one of the largest among the RNA viruses. They codify for four structural proteins—five in the case of some BCoV—and several non-structural proteins comprised mainly on two ORFs (ORF1a and ORF1b) that will eventually be cleaved into 15 or 16 proteins (Desforges et al., 2014b). It has been described that non-structural proteins are the leading cause of host immune system modulation and they also play a role in the replication of the genetic material of the virus (Gorbalenya et al., 2006). As described in mice, viral entry is mediated through the interaction of viral Spike (S) protein and cellular CECAM-1 receptor, along with other co-receptors (Williams et al., 1990; Bergmann et al., 2006). From there, the virus can replicate its RNA and translate it into proteins. Among the cells that are permissive to MHV infection are macrophages, microglia and astrocytes (Bergmann et al., 2006; Jacomy et al., 2006). Remarkably, the year 2006 St-Jean et al. (2006) described the recovery of an infectious HCoV-OC43 with neurovirulent capacities from a full-length cDNA clone inserted in a BAC, with the same phenotype as a WT virus, generating an interesting methodological approach for the study of this virus.

Despite HCoV capacities to infect CNS, it has been recently characterized, its presence in human CNS-related samples date back as early as 1980, where the first detection of this virus was performed in autopsy of patients with MS (Burks et al., 1980). Following that, a few reports confirming the presence of this virus in samples from patients with MS was confirmed through several methods (Murray et al., 1992; Stewart et al., 1992). The year 2000, through research in an autopsy samples from patients with various neurological diseases (being MS most prevalent among them). showed that a 67% were positive for HCoV (with HCoV-229E being twice as common as HCoV-OC43; Arbour et al., 2000). Moreover, the prevalence of OC43 in MS samples was statistically higher than in control patients, is the first report to provide a significant indication of the neurotropic capacity of these respiratory pathogens (Arbour et al., 2000).

**Figure 3:**
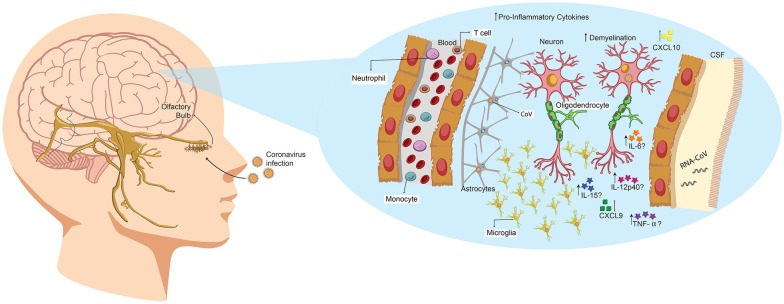
Human coronavirus (HCoV) enters the CNS through the olfactory bulb, causing inflammation and demyelination. Upon nasal infection, HCoV can reach the CNS through the olfactory bulb, as ablation of this part of the brain restricts its neurotropic capacities in mice. Once the infection is set, the virus can reach the whole brain and CSF in less than 7 days. Accordingly, it has been described that this virus can induce demyelination. Likewise, primary glial cultures have been described to secrete IL-6, IL-12p40, IL-15, TNF-α, CXCL9 and CXCL10 upon viral infection.

The first case of SARS-CoV infection with neurological manifestations was reported the year 2003 in a 59-year-old woman (Hung et al., 2003). She was first admitted with swinging fever, chills, productive coughing and diarrhea, which eventually lead to oxygen requirements, vomit, seizures and episodes of four-limb twitching. The respiratory failure continued until she was sedated, and ventilation was required (Hung et al., 2003). SARS-CoV infection was confirmed in both tracheal aspirates and CSF samples, followed by ribavirin treatment, with no improvement in seizures persistence. With additional treatments, seizures were no longer detected, and she was discharged 3 weeks after admission (Hung et al., 2003). The following year, another case of SARS-CoV infection with detection of genetic material in CSF samples was reported in a 32-year-old woman (Lau et al., 2004; Figure 3). Detection was also positive for stool specimens and peritoneal fluids. The patient was admitted in week 26 of pregnancy and at 7 days post-admission, mechanical ventilation was required. At day 8, she presents a sign of acute renal failure, and pregnancy termination was decided. Through cesarean, a baby girl was born without further complications (Lau et al., 2004). At day 22 the patient was still sedated and on mechanical ventilation, with convulsions and loss of consciousness. Starting at day 27 she was extubated and from there followed an uneventful recovery. She exhibited no further convulsions and no evident sequelae (Lau et al., 2004). Organ dissemination of SARS-CoV in autopsy samples from patients that died of this disease was determined. The report indicates the presence of SARS-CoV-N protein and viral RNA in the stomach, small intestine, kidney, sweat glands, parathyroid, pituitary gland, liver and cerebrum, further confirming the capacity of this virus to induce a systemic infection (Ding et al., 2004).

A case report of HCoV-OC43 detection in nasopharyngeal and CSF samples from a child patient was performed the year 2004 (Yeh et al., 2004). The child exhibited acute disseminated encephalomyelitis, a low-prevalence CNS disease that induces demyelination, being this the first case related with HCoV. No other infectious agents were detected in any of the samples (Yeh et al., 2004). Following this, a brief characterization of the cytokine profile in the CNS, induced by SARS-CoV-infection, was published. Therein, the authors indicated that both chemokine induced by IFN-γ (CXCL9, a CXC chemokines family member) and IFN-γ-inducible protein 10 (CXCL10) were highly induced in brain samples from a deceased patient (Xu et al., 2005; Figure 3). However, there are reports about high levels of CXCL10 in SARS-CoV infected patients, with no neurological manifestations. Therefore the authors suggest that CXCL9 could be closely related to CNS infection (Xu et al., 2005).

A recent report—performed the year 2016 by Li et al. (2016)—describes several features of HCoV-infection in either respiratory and CNS infection. From the 183 children hospitalized by suspected acute encephalitis, a total of 22 were positive for HCoV infection, showing vomiting, headache and fever as the most recurrent symptoms among them. Remarkably, they also showed there that peripheral blood lymphocytes and eosinophils counts in CNS-HCoV-infected patients were lower when compared with respiratory-HCoV-infected patients, while the opposite trend was observed for neutrophils (Li et al., 2016). These differences in the recruitment of immune cells could be related to the immune response elicited by the virus, either it is respiratory-restricted or exhibits neurotropism capabilities (Li et al., 2016).

HCoV capacity to reach CNS after the nasal infection has been described previously in mice, particularly for HCoV-OC43 (St-Jean et al., 2004). St-Jean et al. (2004) reported that upon infection, viral antigens are detected in the olfactory bulb 3 days later, with no presence of virus in perivascular blood cells or any other part of the brain. After 7 days, the virus is detected throughout the whole brain tissue, indicating that it can rapidly propagate once set in CNS. This replication leads to a rapid death by acute encephalitis of infected mice. Remarkably, ablation of the olfactory bulb prevented the spread of mouse hepatitis virus (MHV), upon nasal infection (Perlman et al., 1990). Therefore, HCoV exhibits an intrinsic capability to infect neural cells and spread from CNS to the periphery via a transneural route, as has also been seen for MHV (Perlman et al., 1990; Barthold et al., 1990; Figure 3).

Mice studies are mainly performed with MHV, a virus that belongs to the BCoV genus and is genetically related to HCoV-OC43; likewise, the disease at CNS as elucidated by both viruses are similar, as they both induce demyelination (Jacomy and Talbot, 2003; Bergmann et al., 2006; Figure 3). Jacomy and Talbot (2003) were among the first to describe a mouse model to characterize the CNS disease in their publication the year 2003. Therein, they exhibit that BALB/c and C57BL/6 mice could be infected through nasal instillation with MHV, although they chose to use intracerebral inoculation to favor CNS infection (Jacomy and Talbot, 2003). They also determined that viral RNA could be detected in brain, heart, spleen, lungs, liver and muscles (Jacomy and Talbot, 2003). Likewise, the year 2004 Glass et al. (2004) described a systemic non-lethal model of infection for SARS-CoV in C57BL/6 mice that eventually reached the brain. Finally, in Jacomy et al. (2006) described that HCoV-OC43 could infect glial and neuronal cells of both rat and mice (Figure 3). Therein, they also showed that surviving animals exhibited decreased motor functions. Recently, Wheeler et al. (2018) described that microglia is essential for the regulation of MHV infection, as depletion of this cell type led to faster viral replication, enhancing its capacity to avoid adaptive immunity. According to this, glial primary cultures of MHV-A59-infected cells showed an increase in the secretion of IL-12 p40, TNF-α, IL-15 and IL-6 compared with a non-neurotropic MHV, suggesting that the infection with a neurotropic virus activates glial cells and induces a pro-inflammatory state (Li et al., 2004; Figure 3).

As described so far, CoVs are respiratory viruses that exhibit neurotropic capacities that not only allows them to achieve latency and avoid the immune response of the host, but also have neurological implications that can complicate the disease associated to its infection. Although their mechanisms and routes to reach the CNS have not been elicited yet, the detection of either viral proteins or genetic material in this issue has been confirmed thoroughly, branching the researcher’s goals into acquiring new insights regarding this topic. So far, epidemiological reports have allowed to achieve this, but further work in animal models is required to fully comprehend the mechanisms that CoV uses to reach CNS and to achieve more suitable treatments to resolve this viral infection without an exacerbated disease.

## Human Metapneumovirus

The hMPV is a new virus first reported the year 2001 in Netherlands (van den Hoogen et al., 2001), and since its discovery, several epidemiological reports have placed it among the most prevalent respiratory viruses worldwide, although its disease burden has not been thoroughly characterized (Hamelin et al., 2006; Edwards et al., 2013). It is responsible for respiratory illness mainly in newborns, infants and immunocompromised patients, although it can also infect healthy adults, with mild symptoms (Edwards et al., 2013). Its genome is negative sense and about 13 Kb length, with nine structural proteins codified on it (van den Hoogen et al., 2002; Schildgen et al., 2011). hMPV belongs to the *Mononegavirales* order, *Pneumoviridae* family and the *Metapneumovirus* genus (Amarasinghe et al., 2018; King et al., 2018). Since it is closely related to hRSV, both in its classification and its genome, their diagnosis has been usually mistaken (Edwards et al., 2013). Currently, there are no effective treatments against this virus, neither vaccines nor specifics treatments, mainly due to the lack of thorough knowledge associated with its immune response and disease pathogenesis (Zlateva and Van Ranst, 2004; Schildgen et al., 2011).

In humans, there is a handful of reports associated with encephalitis and hMPV-infections. The first approach to this topic was a report described the year 2003 in China (Peiris et al., 2003). Therein, the authors describe several cases of children with acute respiratory diseases, particularly 587 patients, of which 32 were positive for hMPV RNA detection (Peiris et al., 2003). Although the scope of this report was not to associate hMPV with any CNS abnormality, they do report five cases of children with a febrile seizure, reaching levels comparable to the ones seen in influenza, the respiratory viruses most prone to induce seizures, as they state (Peiris et al., 2003). Following this, the first case in which the presence of hMPV RNA in brain samples of a patient with encephalitis was reported, was the year 2005 in Germany (Schildgen et al., 2005). This report described the case of a 14-month-old boy that was received in a primary care hospital with a high fever and unresponsiveness to stimulus, either verbal or tactile. Previous to its internalization, during the same day he reported seizures and no spontaneous eye movement (Schildgen et al., 2005). After 10 days of hospitalization without further improvement, the child was considered to be dead and was extubated. Autopsies revealed the presence of genetic material of hMPV in both brain and lungs, and no other virus, such as hRSV or HSV, were detected (Schildgen et al., 2005). Concomitantly the same year, a case of encephalitis where hMPV RNA was detected in nasal mucus and tracheal aspirates was reported (Kaida et al., 2006).

From this point on, several cases were described where hMPV-infection was related to encephalitis, and in some cases, the detection of genetic material in different CNS samples was detected. The year 2007, the death of a 6-month-old girl was reported 9 days after her admission to a healthcare unit (Hata et al., 2007). She exhibited generalized convulsions and was diagnosed with acute encephalopathy the day she was admitted, and 24 h later she fell into a coma. Although there was no detection of genetic material in CSF, the presence of hMPV-F protein was confirmed by RT-PCR in throat swab and urine (Hata et al., 2007). Two years later, Arnold et al. (2009) described in an epidemiological report the presence of nine cases of hMPV-infection related to different spectrums of CNS abnormalities, distributed in two different study groups. In the first group, composed of 1474 patients, 76 samples were hMPV positive, and of those, four patients were reported with seizures. Therein, they also expose data regarding hRSV, showing that 145 of the samples were positive for this virus and only one of the patients was reported with seizures. Through statistical analysis, they can confirm that the frequency of hMPV patients with seizures is statistically significant, unlike the one has seen in hRSV. The second group consisted solely of patients hospitalized with high fever or any CNS abnormality, ranging from the age of 6-month-old to 18 years old. In that group, they reported five patients with hMPV, where seizures were diagnosed in three (Arnold et al., 2009). They also described another patient with hMPV-infection and detection of genetic material of enterovirus in CSF. Remarkably, the presence of hMPV genetic material was not detected in the CSF of any of the available samples. Despite this, they indicate that a normal CSF profile does not necessarily exclude a neurotropic mechanism, as seen for rabies encephalitis, in which only half of the CSF samples are positive for CSF pleocytosis (Arnold et al., 2009). Likewise, the case reported above by Schildgen et al. (2005) identified hMPV RNA postmortem, despite normal CSF cell count and a negative detection of the virus by PCR in spinal fluid (Arnold et al., 2009).

On the same line, the year 2012 the first report associated with the hMPV genetic material in CSF was published (Sánchez Fernández et al., 2012). Sánchez Fernández et al. (2012) confirmed this detection through PCR—in a 10-year-old girl with signs of acute encephalitis—and then characterized the evolution of the disease and also included neuroimaging features therein. Detection of other etiological agents, such as herpes virus and adenovirus, were negative. Magnetic resonance imaging (MRI) showed signs of acute encephalitis and demyelinating process mainly in the temporal and occipital lobes, but these lesions eventually spread to frontal and parietal lobes (Sánchez Fernández et al., 2012). Eventually, thanks to the treatment received, clinical improvement was noted and—35 days post hospitalization—she was discharged home, unlike many other cases, where these symptoms resulted in the death of the child (Sánchez Fernández et al., 2012). However, some of the sequelae registered include inappropriate social abilities and infantile behavior, with severe attention—but no motor or memory—deficits (Sánchez Fernández et al., 2012).

More cases in which status epilepticus was reported—a single long-lasting seizure or several seizures in a specific time range- were associated with hMPV. A 3.5-year-old girl with hMPV respiratory disease was reported the year 2013 (Niizuma et al., 2014). The year 2014, two cases were reported, a 15-month old and a 18-month-old girl, that reported hMPV infection and eventually developed respiratory failure (Webster et al., 2014). These three cases were discharged without apparent sequelae. The year 2015, the first reported case of a child with refractory status epilepticus—a non-responsive condition with a worse prognosis than commonly responsive status epilepticus—was described (Vehapoglu et al., 2015). The 4-month-old boy was received and eventually transported to pediatric ICU. CSF PCR analysis was negative for HSV, and nasal scraps were only positive for hMPV. As stated above, treatment with antiepileptic drugs resulted in unresponsiveness. Eventually, seizures passed, and 25 days post-admission she was discharged. No evident sequelae were detected, though antiepileptic drugs were maintained for the following 6 months (Vehapoglu et al., 2015).

Most recently, cases of adults with acute encephalitis and hMPV detection have been described. The first report was published the year 2015 in Australia, in a 47-year-old man (Fok et al., 2015). The man was found unconscious after 2 days of respiratory symptoms and immediately hospitalized. PCR testing of CSF elicited no presence of classical CNS pathogens (HSV, varicella zoster virus, enterovirus) and eventually nasopharyngeal aspirates were positive for hMPV and no other respiratory viruses, although at this point, not enough CSF was left for further testing. No evident sequelae were detected during the following months (Fok et al., 2015). Remarkably, lesions found in the MRI analysis were similar to the ones described in the previously reported cases (Schildgen et al., 2005; Sánchez Fernández et al., 2012). Then the year 2017, two reports—one associating hMPV respiratory infection, the other one indicating the presence of hMPV genetic material in CSF—were published. Early during that year, Jeannet et al. (2017) described the case of a 61-year-old Swiss man with influenza-like symptoms, which eventually suffered from a headache and seizures. MRI was inconclusive, and PCR analysis was negative for CSF samples, although nasopharyngeal swabs were positive for hMPV and no other respiratory virus (Jeannet et al., 2017). Then, later that year, the case of a 32-year-old man was reported, although the case dated from 3 years before its publication. The man was admitted with unspecific low backache and fever, MRI was normal, and no unusual symptoms were detected (Tan and Wee, 2017). Eventually, the patient de-saturated and respiratory failure was diagnosed, with posterior mechanical ventilation. Bronchoalveolar lavages and CSF PCRs were both positive for hMPV (Tan and Wee, 2017). The patient was treated for 1 week with ribavirin (unspecific antiviral) and eventually respiratory symptoms subdued, although he exhibited reduced cognition and intermittent agitation as sequelae. He began rehabilitation, but there was not a significant improvement throughout the following 6 months (Tan and Wee, 2017).

Remarkably, no studies have been performed describing the ability of hMPV to cause CNS damage in mice. However, it has been described that hMPV can persist in the lung of mice after acute infection (Alvarez et al., 2004; Liu et al., 2009). This characteristic could be aiding the virus to eventually reach the CNS, as the latency state is achieved through the infection of neuronal processes that innervate the lungs, as the genetic material of the virus can be detected in them (Liu et al., 2009). Moreover, as described by the authors, latent virus can be reactivated upon treatment with immunosuppressive drugs such as steroids.

hMPV remains a viral agent that needs to be thoroughly characterized, as although most of its respiratory-related pathogenesis has been taken to spotlight, its capacity to achieve latency in CNS cells is yet to be elicited. Although there are extensive case reports that indicate neurological manifestations associated to hMPV-infection in humans, further studies are required in mice models to characterize this disease. Considering that this virus was first described only 17 years ago, there is plenty of work to be done in order to fully comprehend the impact that this virus may be playing in CNS-related pathologies.

## Concluding Remarks

Respiratory viruses are the leading cause of bronchiolitis and pneumonia throughout the world, affecting varying ranges of ages, but being more aggressive in children, elderly and immunocompromised individuals. Most prominent respiratory viruses are hRSV, IV, CoV and hMPV. Remarkably, extra-pulmonary symptoms have been described in these viruses, highlighting their capacity to cause neurological complications. Febrile seizures, loss of consciousness, convulsion, ataxia, status epilepticus, encephalitis, myelitis, neuritis and MS are among the several extra-pulmonary symptoms that have been described. Case reports of children, elderly and even adults exhibiting these symptoms have been described throughout the years for all of these viruses, bringing to spotlight the urgency to describe their neuroinvasive capacity. Moreover, the detection of genetic material and even viral proteins in CNS samples, such as CSF or brain, is a recurrent fact described in several case reports.

Several ways to achieve CNS have been described, including transneural and hematogenous pathways. However, the specific mechanisms responsible for dissemination of each of these viruses with neurotropic capacities into the CNS, have not been thoroughly characterized yet. For instance, hRSV has been reported to reach CNS via the hematogenous pathway, although other routes cannot be discarded entirely. IV can reach the brain via the transneural route, mainly by retrograde axonal transport, reaching first vagal and trigeminal nerves. Likewise, CoV has been described to reach the brain via olfactory bulb, spreading from this point onward into the CNS and the periphery. Since hMPV is an emerging virus, no studies have been performed regarding its capacity to reach CNS, so further data for this virus is required.

So far, several mice models have been established for every one of these viruses, allowing the acquisition of new data and the development of new insights of their neurotropic capacities and their neurological manifestations. However, further researches are still required, as many aspects of these CNS pathologies remain unknown. These studies will add new approach edges to a topic that urgently needs to be characterized, as many children may currently be exhibiting these symptoms and are not being treated properly, as the respective viral infection may not be diagnosed.

## Author Contributions

All authors listed have made substantial, direct and intellectual contribution to the work and approved it for publication.

## Conflict of Interest Statement

The authors declare that the research was conducted in the absence of any commercial or financial relationships that could be construed as a potential conflict of interest.
